# Pulmonary Edema in COVID-19 Patients: Mechanisms and Treatment Potential

**DOI:** 10.3389/fphar.2021.664349

**Published:** 2021-06-07

**Authors:** Xinyu Cui, Wuyue Chen, Haoyan Zhou, Yuan Gong, Bowen Zhu, Xiang Lv, Hongbo Guo, Jinao Duan, Jing Zhou, Edyta Marcon, Hongyue Ma

**Affiliations:** ^1^ Jiangsu Collaborative Innovation Center of Chinese Medicinal Resources Industrialization, and Jiangsu Key Laboratory for High Technology Research of TCM Formulae, College of Pharmacy, Nanjing University of Chinese Medicine, Nanjing, China; ^2^ Donnelly Centre for Cellular and Biomolecular Research, University of Toronto, Toronto, ON, Canada

**Keywords:** COVID-19, pulmonary edema, abnormal lung humoral metabolism, syndrome coronavirus 2, drug, traditional Chinese medicine

## Abstract

COVID-19 mortality is primarily driven by abnormal alveolar fluid metabolism of the lung, leading to fluid accumulation in the alveolar airspace. This condition is generally referred to as pulmonary edema and is a direct consequence of severe acute respiratory syndrome coronavirus 2 (SARS-CoV-2) infection. There are multiple potential mechanisms leading to pulmonary edema in severe Coronavirus Disease (COVID-19) patients and understanding of those mechanisms may enable proper management of this condition. Here, we provide a perspective on abnormal lung humoral metabolism of pulmonary edema in COVID-19 patients, review the mechanisms by which pulmonary edema may be induced in COVID-19 patients, and propose putative drug targets that may be of use in treating COVID-19. Among the currently pursued therapeutic strategies against COVID-19, little attention has been paid to abnormal lung humoral metabolism. Perplexingly, successful balance of lung humoral metabolism may lead to the reduction of the number of COVID-19 death limiting the possibility of healthcare services with insufficient capacity to provide ventilator-assisted respiration.

## Introduction

COVID-19, an infectious disease caused by a severe acute respiratory syndrome coronavirus 2 (SARS-CoV-2), has a global reach. By March 31st of 2021, a total of 128.54 million cases of severe Coronavirus Disease (COVID-19) have been diagnosed globally, and a total of 2.81 million people have died from the disease (WHO, 2021). Currently, no specific drug has been developed to against COVID-19, although some existing drugs have been repurposed and approved for treating hospitalized patients (Ferner and Aronson, 2020; Schlagenhauf et al., 2020). Recently, several companies came out with vaccines against COVID-19 which have been approved for use. Some others will likely be approved soon (Kaur and Gupta, 2020; Thanh Le et al., 2020). However, it is still unclear how the vaccination will proceed and how fast can vaccinations be done. In the meantime, we urgently need potent reduction in fatality of COVID-19.

Pulmonary edema is the disequilibrium between formation and reflux of lung tissue fluid leading to the absorption of massive tissue fluid by lung lymph and vein failure. The fluid transudes into and accumulates in the interstitium of lungs and finally alveolars from lung capillary, leading to severe disorder of pulmonary ventilation and gas exchange (Staub, 1974). In COVID-19 patients, pulmonary edema is diagnosed by lung ultrasound and a computerized tomography (CT) scan (Udugama et al., 2020). The condition presents itself as a slowly evolving pneumonia with insidious early onset interstitial pulmonary edema that undergoes acute exacerbation in the late stages and alveolar edema (Xu et al., 2020; Wiersinga et al., 2020). Currently, these symptoms are the primary consequences of pulmonary virus infection. It is known that SARS-CoV-2 invades human cells by binding angiotensin-converting enzyme-2 (ACE-2) receptor and other membrane ectopeptidases (Xu et al., 2020). When there, the virus itself and virus-mediated protein-protein interactions lead to the lung inflammatory storm responsible for the observed increasing vascular permeability in lung and pulmonary edema (Tang et al., 2020). It is likely that alveolar fluid clearance (AFC) failure plays a major role in the pathogenesis of pulmonary edema. The imbalance of fluid metabolism, pulmonary fluid clearance (PFC) and rich-protein fluid entrance, may be a key reason for the acute exacerbation of pulmonary edema in COVID-19 patients.

Here, we describe molecular mechanisms of PFC and propose that proteins functioning in this process might serve as an underappreciated, but yet promising targets for reducing lung edema in severe COVID-19 patients. These proteins include ion channels (Na channel, K channel and TRPV4), aquaporins (AQP), renin angiotensin system (RAS) proteins, and bradykinin/hyaluronic acid-related enzymes. Drugs targeting at least some of these proteins have already existed and could be repurposed to manage pulmonary edema seen in SARS-COV-2 infections. Chinese Medicine (TCMs), already widely used in China, may also be beneficial in addressing pulmonary edema in COVID-19 patients (Zhang et al., 2020; Yang et al., 2020d). There are also several natural compounds which were previously shown to have positive effects on the lung edema-associated targets described in this paper (Zhou et al., 2006; Ho et al., 2007; Ji et al., 2014; Wang et al., 2015; Qu, 2019; Fan et al., 2020; Lung et al., 2020). In this review, we discuss the clinical characteristics of COVID-19, current as well as potential new treatments based on the reduction of lung edema through various means, drugs, TCMs or natural compounds. We speculate that treatment of lung-edema will lead to a lower mortality in COVID-19 patients with severe infections.

## Virological Characteristics of COVID-19

**FIGURE 1:**
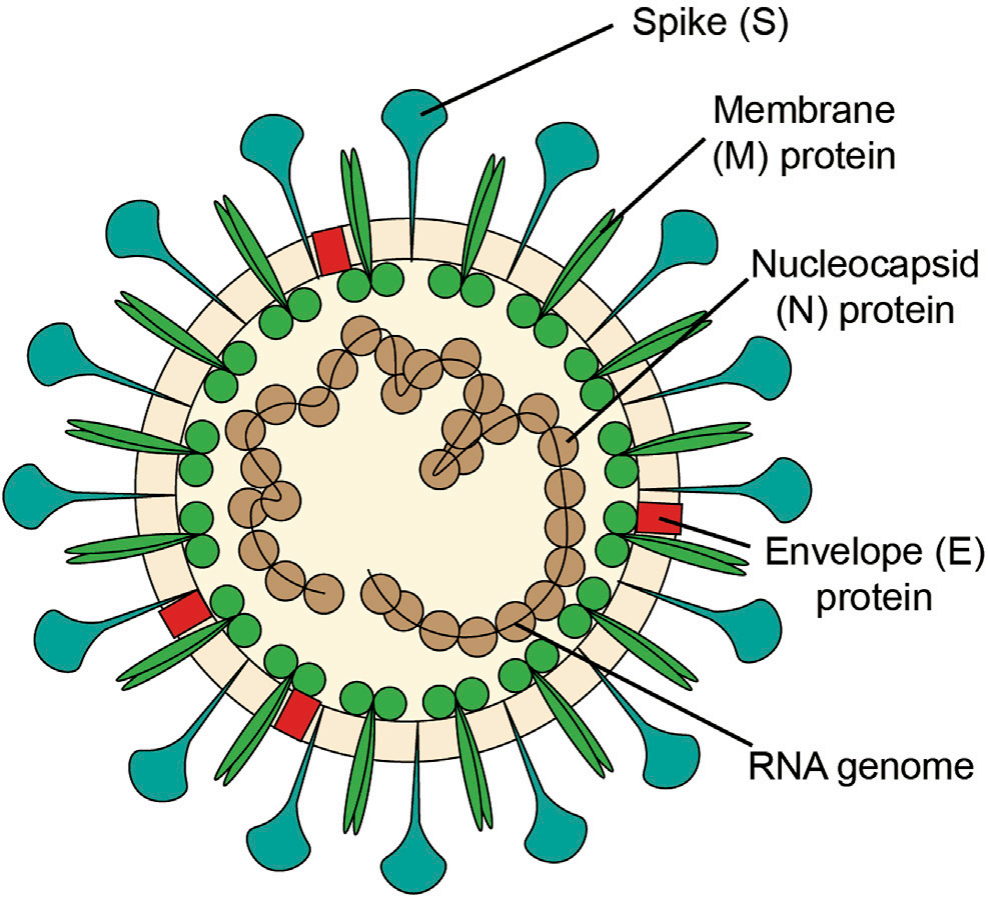
A vision of coronavirus with the minimal set of structural proteins.

SARS-CoV-2 is an enveloped RNA coronavirus of the genus β, and is the seventh coronavirus which can infect human (Xu et al., 2020). The structure of coronavirus (Figure 1 A vision of coronavirus with the minimal set of structural proteins.) includes glycoproteins, membranes and nucleic acids. The spike (S) protein of coronavirus, one of the surface glycoproteins, is divided into two functional units, S1 and S2. S1 facilitates virus infection by binding to host receptors, and S2 regulates the membranes fusion to enable viral RNA entering into host cells for further replication. Therefore, the S protein determines the host cell of the virus, regulates the viral attachment and fusion with the host cell membrane, and promotes cellular invasion. As such, the S protein is essential for viral infection (Heald-Sargent and Gallagher, 2012; Li, 2016; Walls et al., 2020).

**FIGURE 2:**
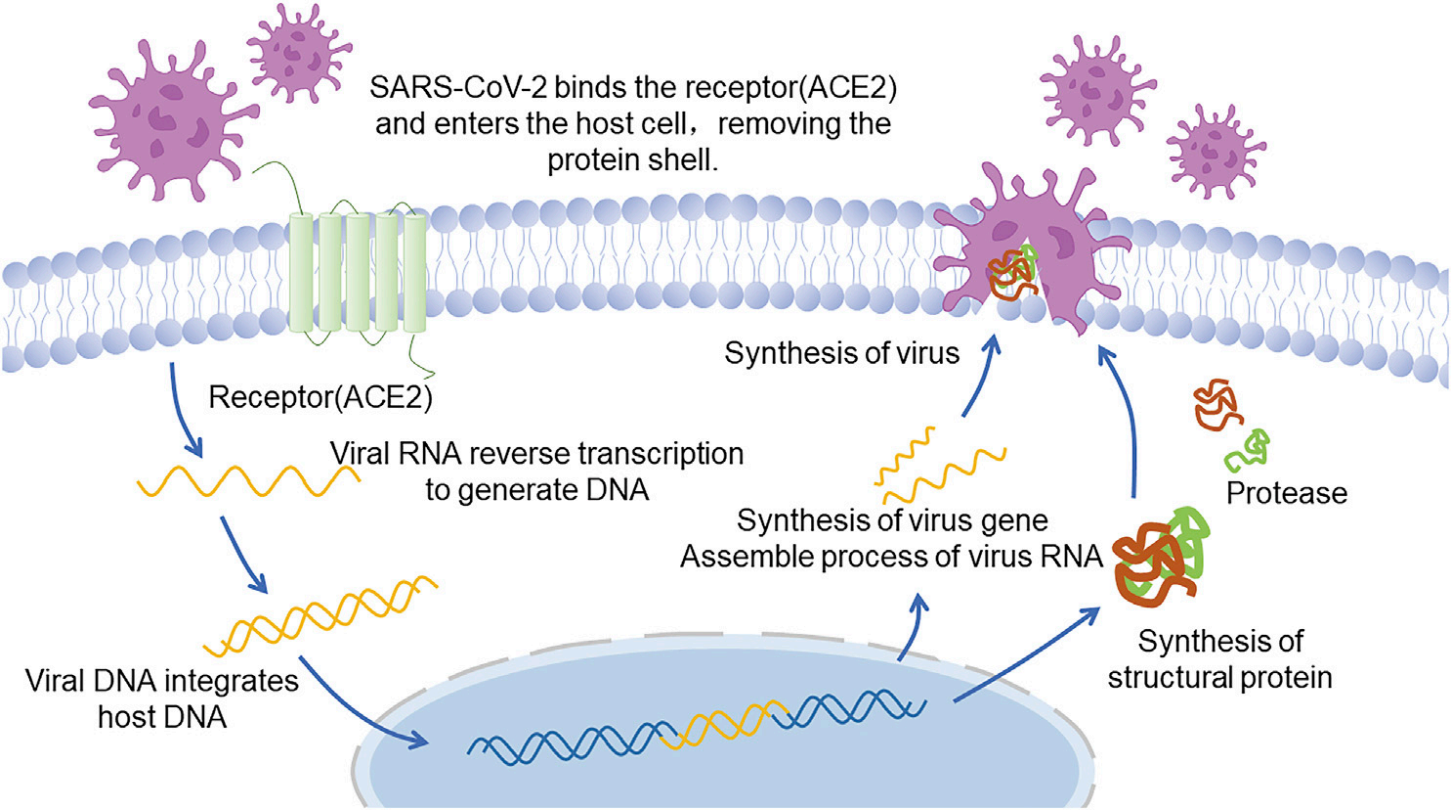
Infection and replication process of SARS-CoV-2.

It has been shown that SARS-CoV-2 infects human cells via specific binding of S-protein to angiotensin-converting enzyme 2 (ACE2) (Figure 2 Infection and replication process of SARS-CoV-2) (Xu et al., 2020) and the binding affinity between these two proteins is 10–20 times greater than that of SARS-CoV and ACE2 (Yan et al., 2020; Wrapp et al., 2020). ACE2 is most abundantly expressed in human vascular endothelial cells as well as alveolar and intestinal epithelial cells. It is also highly expressed in cardiomyocytes, epithelial cells of renal proximal convoluted tubule, urothelial cells, esophagus, and ileum (Harmer et al., 2002; Zhang et al., 2020), facilitating a quick invasion of the human body by SARS-COV-2 and causing complications. The latest research revealed that alveolar macrophages, which normally play a protective role, may also be infected by SARS-CoV-2 and release T cell chemokines, resulting large amounts of T cells gathering in lung and generating IFNγ. IFNγ will continually induce inflammatory cytokines released by alveolar macrophages, promoting the activation of T cells and forming a positive feedback loop that drives persistent alveolar inflammation (Grant et al., 2021). It is worth mentioning that, except alveoli, which is widely known as the target tissue, cardiomyocyte can be infected as well. Researches proved that SARS-CoV-2 can directly infect human induced pluripotent stem cell-derived cardiomyocytes (hiPSC-CMs) as well as an engineered heart tissue (EHT), in cellular and organ level respectively, suggesting that the virus can replicate rapidly in the cardiomyocytes, infecting other cardiomyocytes, contributing to cardiomyocyte cell death, myocardial inflammation and even heart failure (Sharma et al., 2020; Bailey et al., 2021).

## Abnormal Lung Humoral Metabolism in COVID-19

**FIGURE 3:**
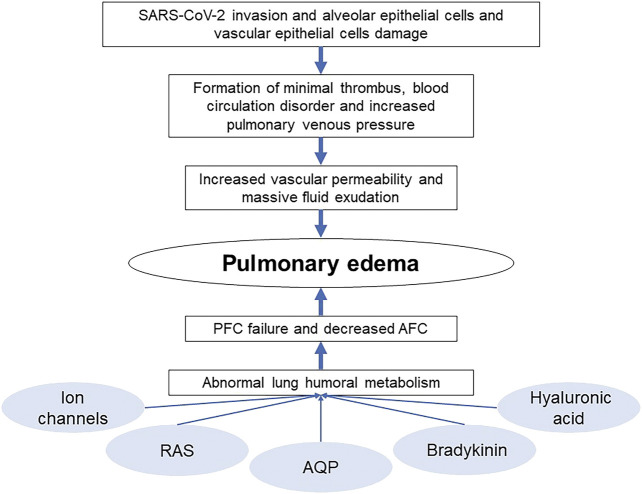
Cause of COVID-19 pulmonary edema.

SARS-CoV-2 invasion leads to alveolar and vascular epithelial cells damage impelling the formation of minimal thrombus, increasing pulmonary venous pressure and vascular permeability and leading to massive loss of tissue fluid. Besides those direct causes of COVID-19 pulmonary edema, there are other factors that can be described as abnormal humoral metabolism which can influence the AFC and PFC, resulting the manifestation of pulmonary edema (Figure 3 Cause of COVID-19 pulmonary edema).

Abnormal humoral metabolism is mainly manifested as imbalances of water and electrolytes. Water and sodium disturbances along with the unusual serum potassium levels are most common. It has been shown that the incidence and severity of COVID-19 are closely related to abnormal metabolism of inorganic salts. Serum sodium shows a decrease trend in CODIV-19 development (Krolicka et al., 2020). Hyponatremia (low blood sodium concentration) as well as low concentrations of potassium and calcium in the blood serum are also associated with COVID-19 (Lippi et al., 2020). The functional inhibition of relevant lung enzymes and ion channels may disturb AFC, thus resulting pulmonary edema in COVID-19 patients. Na^+^/K^+^-ATPase and ion channels (sodium, potassium, AQPs, and TRPs) are all involved in the regulation of AFC.

### Clinical Characteristics and Pathological Mechanism of COVID-19

COVID-19 patients often have pathological features such as pulmonary interstitial or alveolar edema, diffuse tracheal phlegm thrombus and pulmonary inflammatory lymphoid infiltration, and are prone to acute respiratory distress syndrome (ARDS), causing lung injury (Cutts et al., 2017). The clinical manifestations and CT scans show the presence of ARDS in critical COVID-19 patients (Ai et al., 2020; Guan et al., 2020; Huang et al., 2020). Some patients also have leucopenia and lymphopenia, suggesting a weak immune function, and high prothrombin time and D-dimer level, indicating abnormal blood clotting function (Wang et al., 2020), which can all lead to lung damage and the severer pulmonary edema. Patients in intensive care units (ICU) have higher plasma levels of IL2, IL7, IL10, MCP1, MIP1A, GSCF, IP10, and TNFα (Huang et al., 2020). In these patients, white blood cells count, neutrophil count and D-dimer level keep rising while lymphocyte count keeps decreasing as the disease progresses. Therefore, the infection along with the rapid replication of SARS-CoV-2 causes a large amount of body fluid permeating through pulmonary alveoli, leading to ADRS. As the infection progresses, the immune function is impaired, causing damage to multiple organs, additional complications, and eventually death (Yang et al., 2020).

### Sodium Channels and Sodium Pumps

**FIGURE 4:**
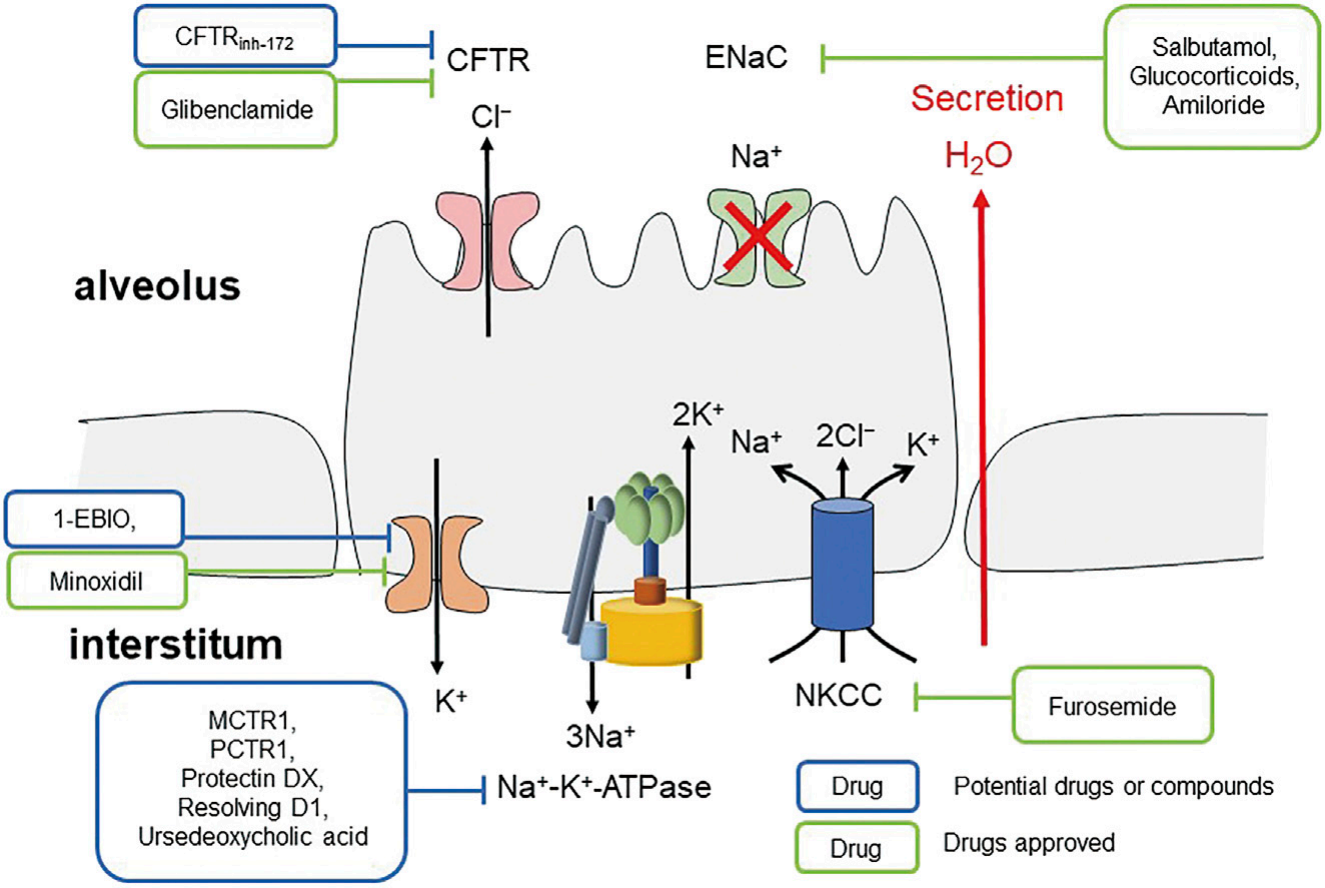
Mechanism of inhibiting ENaC inducing pulmonary edema.

Sodium transport is the main ion transport involved in the AFC. Epithelial sodium channel (ENaC), present in human lungs, kidneys and other organs, plays a vital role in lung fluid clearance (Figure 4 Mechanism of inhibiting ENaC inducing pulmonary edema) (Matthay et al., 2002). Its active absorption of Na+ is the main driving force of fluid clearance at birth and alveolar fluid absorption at adult stage (Bardou et al., 2012). The cystic fibrosis transmembrane conductance regulator (CFTR) and the ENaC located in the airway apical membranes and alveolar epithelial cells are essential in regulating lung fluid balance across airway as the chloride (Cl^−^) and bicarbonate (HCO3^−^) secretion conduits, and alveolar epithelia by sodium (Na^+^) ion absorption (Matalon, 1999; Birket et al., 2016; Londino et al., 2017). These channels are important in maintaining the optimum volume and ion constitution of bronchial periciliary fluid and alveolar lining fluid layers, which are necessary in appropriate pathogens mucociliary clearance and optimum gas exchange, respectively (Londino et al., 2017). Therefore, severe infections, which are induced by influenza virus, target the distal lung epithelial cells, inhibit the ENaC via activating protein kinase C (Kunzelmann et al., 2000), and damage the pulmonary surfactant (Hofer et al., 2015; Ito et al., 2015; Woods et al., 2015). SARS-CoV-2 may act in a similar way. With the presence of active basolateral Na^+^-K^+^-ATPase, inhibition of the entry of apical Na^+^ can generate a concentration gradient inducing the uptake of basolateral Na^+^ with Cl^−^ by Na^+^-K^+^-2Cl^−^ cotransporter (NKCC) and thus induce the apical secretion of Cl^−^ (Solymosi et al., 2013). The secretion of alveolar fluid, caused by inhibiting Na^+^ entry, is sensitive to inhibition of CFTR, NKCC, or Na^+^-K^+^-ATPase (Solymosi et al., 2013), suggesting that CFTR, NKCC or Na^+^-K^+^-ATPase inhibitors may have potential in treating pulmonary edema caused by low expression of ENaC. As in lipopolysaccharide-induced acute lung injury, docosahexaenoic acid and its derivatives stimulate AFC through alveolar ENaC, Na,K-ATPase via ALX/cAMP/PI3K pathway (Wang et al., 2014; Zhang et al., 2017). Moreover, lower expression of alveolar Na-K-ATPase promotes pulmonary edema, and when the expression of Na-K-ATPase α1- and α2-subunits decreases, maximal alveolar epithelial fluid clearance is reduced (Looney et al., 2005).

### Potassium Channels

Potassium channels are usually involved in maintaining the reabsorption of Na^+^ and the steady state of electrochemical gradient, ions and body fluids in airway epithelial cells specifically. Potassium channels up-regulate ENaC expression via activating KvLQT1 pathway so as to control AFC (Bardou et al., 2012). In addition, potassium channels can act as oxygen sensors in alveolar epithelium and thus adjust lung function to environmental changes in O_2_ levels (Bartoszewski et al., 2017). As reported, large-conductance calcium-activated potassium channels (BK_Ca_) in alveoli can reduce alveoli opening during hypoxia, detect O_2_ variation, and adjust ion transport and fluid clearance (Jovanović et al., 2003).

### Aquaporins

The abnormal expression of AQP is closely related to the abnormal alveolar fluid metabolism and the subsequent pulmonary fibrosis of COVID-19 patients. AQP-5 protein, present in the apical membranes of AT-I cell of alveolar epithelium, can regulate the transport of water molecules. It promotes the clearance of surplus fluid in alveoli and keeps alveolar space dry (Wittekindt and Dietl, 2019). The expression of AQP-5 is regulated by inflammatory cytokines, like TNF-α, elevated in the plasma of critical COVID-19 patients (Hui and Zumla, 2019). As Towne et al., (2001) reported, AQP-5 expression significantly declined during pulmonary inflammation and edema, and TNF-α decreased AQP5 mRNA and protein expression levels via TNFR1 and NF-κB pathway (Towne et al., 2001).

Idiopathic pulmonary fibrosis is also a risk factor for severe COVID-19 which can be observed in the CT scans of COVID-19 patients (Xu et al., 2020; George et al., 2020). As Gabazza et al., (2004) reported, lung fibrosis is linked to decreased mRNA and protein expression of AQP-5 in the lung. This is supported by the studies of AQP-5 deficient mice where a fibrotic phenotype with increased deposition of extracellular collagen type I was observed in thickened alveolar walls (Gabazza et al., 2004). Therefore, AQP-5 may be a promising drug target for treating abnormal humoral metabolism as well as lung injury caused by COVID-19.

### Transient Receptor Potential Ion Channels

TRP channels are nonspecific cationic channels located throughout the respiratory system (Clapham et al., 2001), where TRPA1, TRPV1 and TRPV4 are the most abundant TRP subtypes (Kaneko and Szallasi, 2014; Steinritz et al., 2018). Evidences for TRPs as medium of lung injury are emerging from studies on various inhalational chemical threats (Achanta and Jordt, 2020). TRPs regulate their functions through sensory neuronal and nonneuronal pathways and play an important role in complicated pulmonary pathophysiologic events, such as increased intracellular calcium levels, recruitment of pro-inflammatory cells, cough reflex, blocked mucus clearance, epithelia integrity disruption, pulmonary edema, fibrosis and so on (De Logu et al., 2016).

TRPA1 distributes on C-fibers throughout the respiratory system (De Logu et al., 2016). The stimulation of TRPA1 can cause coughing, hypersecretion of mucus, rapid shallow breathing as well as bronchoconstriction (Bessac and Jordt, 2008; Birrell et al., 2009), which, if persistent, may cause ARDS and other chronic diseases. TRPV1, expressed in C-fibers of the vagus nerves innervating airways (Cui et al., 2016), has been considered to play a key role in cough reflex and increased airway sensitivity caused by various diseases (Andrè et al., 2009; Couto et al., 2013). It has been reported that infection with a respiratory-associated virus can significantly increase the expression and activity of TRPV1 (Abdullah et al., 2014). TRPV4, expressed in alveolar type I, type II cells and alveolar capillary endothelial cells, has been considered as a crucial regulator of alveolo-capillary barrier integrity (Alvarez et al., 2006; Yin et al., 2008; Goldenberg et al., 2015; Yin et al., 2016). Studies have confirmed that selective TRPV4 activation induces rapid loss of alveolo-capillary barrier function and consequent alveolar edema formation (Alvarez et al., 2006). Indeed, in several preclinical studies, selective TRPV4 inhibition showed efficacy in preventing or attenuating lung edema (Thorneloe et al., 2012). Moreover, it has been revealed that exosomes derived from human adipocyte can inhibit TRPV4-mediated calcium influx and thus protect mice against ventilator-induced lung injury (Yu et al., 2020). As such, TRPV4 inhibition likely has protective and beneficial effect on mucus clearance and pulmonary edema.

### Renin Angiotensin System and Bradykinin

During the infection of SARS-CoV-2, RAS, BK and hyaluronic acid (HA) are all involved in the regulation of AFC and the formation of pulmonary edema (Garvin et al., 2020). RAS, especially several cleavage products of the peptide angiotensin (AGT) along with their receptors, maintains a balance of fluid volume and pressure. For instance, angiotensin II (Ang II) can typically generate vasoconstriction and sodium retention when binding to the AGTR1 receptor and vice versa via the AGTR2 receptor (Garvin et al., 2020). According to previous studies, activation of AT1 receptor inhibits AFC by down-regulating cAMP and dysregulating ENaC expression, leading to Ang II-dependent pulmonary edema and alveolar filling increase (Deng et al., 2012).

**FIGURE 5:**
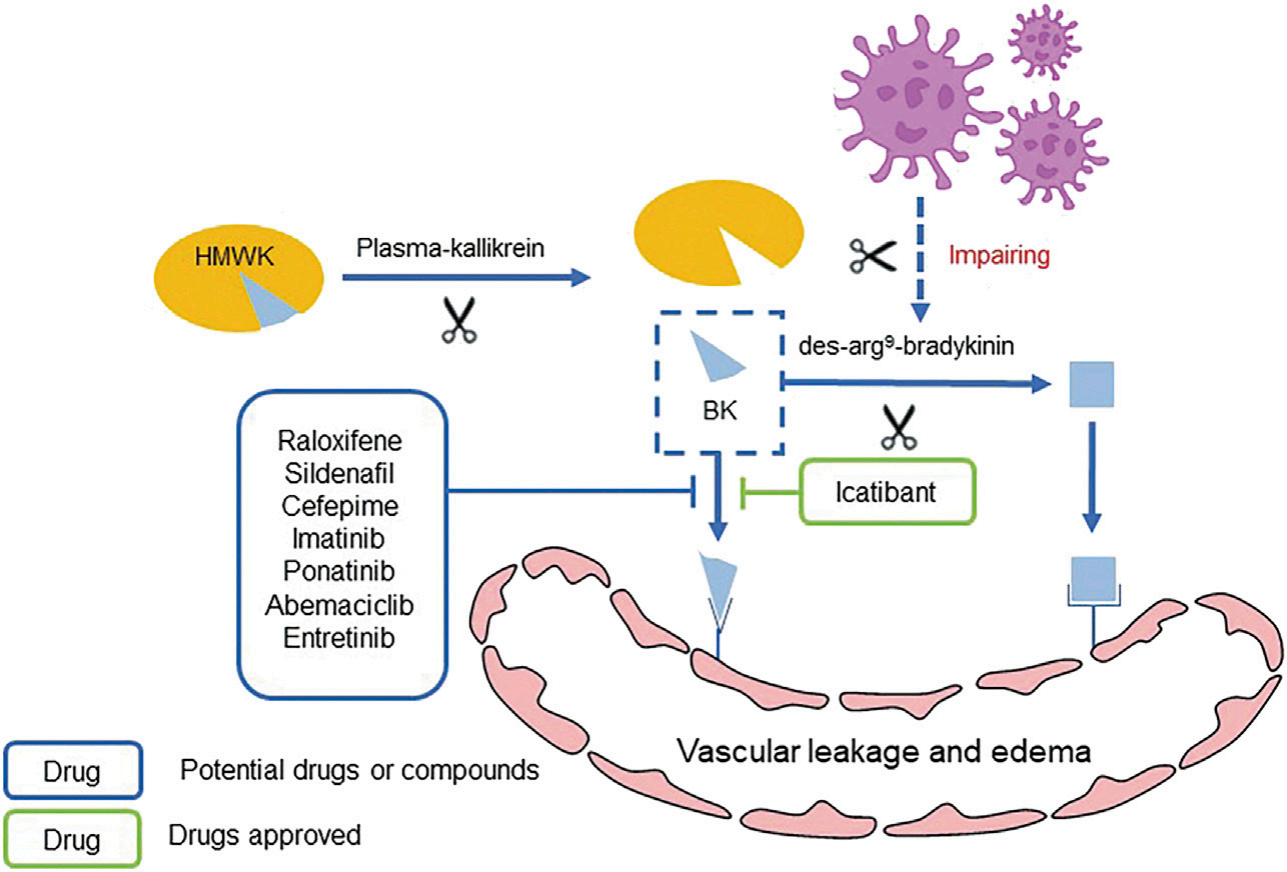
Mechanism of BK inducing pulmonary edema and potential drugs.

Bradykinin (BK) is an important cellular mediator that causes vasodilatation and leaky blood vessels, leading to vascular leakage and edema (de Maat et al., 2020). BK is generated by cleavage of high-molecular-weight kininogen (HMWK) from plasma-kallikrein and binds to the BKB2 receptor, and thus results vascular hemorrhage (Garvin et al., 2020). Inhibition of ACE2 by SARS-CoV-2 impairs the hydrolysis of des-arg^9^-bradykinin. Therefore, the excessive release and decreased hydrolysis of BK through activating BKB1 and BKB2 receptors result in extra vascular leakage and pulmonary edema (Figure 5 Mechanism of BK inducing pulmonary edema and potential drugs) (Zwaveling et al., 2020).

### Hyaluronic Acid and Proteoglycans

CT images of COVID-19 patients revealed fluid and clear liquid jelly in their lungs, both closely linked to HA (Xu et al., 2020; Wang et al., 2020). HA is a polysaccharide existing in most connective tissues which can trap approximately 1,000 times its weight in water and form hydrogel. HA-related hydrogel has been found in both, ARDS and SARS. Inflammatory cytokines and inflammatory storm in COVID-19 patients can strongly induce the expression of HA-synthase-2 (HAS2), while hyaluronidase level decreases, resulting in the accumulation of HA and inducing ARDS and pulmonary edema (Shi. et al., 2020).

In addition, HA is a part of a three-dimensional matrix in pulmonary interstitial, which consists of HA, PGs and fibrillar macromolecules providing resistance to tissue compression and interstitial fluid expansion (Negrini et al., 2008).When PGs and HA interact with collagen IV, a fibrillar macromolecule modulating capillary permeability in the vascular basement membrane, the compound substance limit fluid influx into the interstitium. Thus, PGs play a key role in the formation of pulmonary edema. The integrity of PG molecules in the vascular capillary basement membrane can make sure that endothelial permeability to fluid and solutes in a low level. However, the activation of matrix metalloproteinases (MMPs), which may be triggered by inflammatory factors (Shapiro, 2001), brings PG degradation, inducing pulmonary edema. It has been observed that MMP-2 and MMP-9, two most crucial MMPs in the lung are over-expressed in pulmonary edema (Negrini et al., 1996; Negrini et al., 1998; Passi et al., 1998), suggesting that MMPs may become a potential target for pulmonary edema treatment.

**FIGURE 6:**
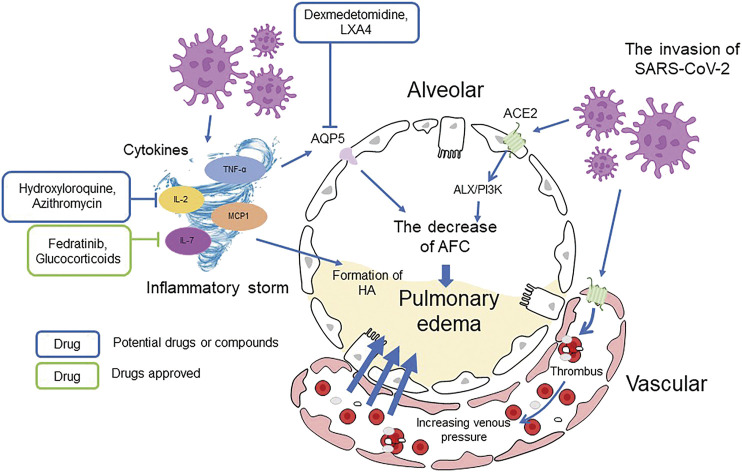
The general regulation approaches of AFC.

Overall, the decreased expression of alveolar Na-K-ATPase, misregulation of sodium, potassium, AQP, and RAS channels and abnormal metabolism of BK and HA can all lead to lung liquid clearance failure and pulmonary edema, resulting in severe lung damage and ARDS in COVID-19 patients (Figure 6 The general regulation approaches of AFC).

## Conventional Treatment of Pulmonary Edema in COVID-19 Patients

Currently, many clinical trials are in progress to test coronavirus treatment, including new drugs and drug repurposing or repositioning. Immune-modulatory agents, supportive cares, and antiviral drugs have been tested as COVID-19 treatments in patients with severe infections (Pascarella et al., 2020; Ren et al., 2020).

Immune-modulatory agents for COVID-19 include tocilizumab, human immunoglobulin and the convalescent plasma. IL6 monoclonal antibody or tocilizumab was thought to work by interrupting inflammatory storm after the infection, but the latest clinical study published in NEJM showed that Tocilizumab was not effective in preventing intubation or death in mild hospitalized COVID-19 patients (Stone et al., 2020). Convalescent plasma have been initially shown to be beneficial for COVID-19 patients with severe infection stabilizing the immune system (Shen et al., 2020), but the subsequent randomized controlled trial did not show significant improvement within 28 days (Li et al., 2020).

Supportive cares for COVID-19 include respiratory support and circulatory support. Patients receive high-flow nasal cannula (HFNC), non-invasive ventilation (NIV), mechanical ventilation or ECMO as respiratory supports (Guo et al., 2020; Pascarella et al., 2020), a crystalloid fluid to ensure body fluid equilibrium (Christ-Crain et al., 2020), and anticoagulants for restraining the thrombus formation to aid in circulatory support (Connors and Levy, 2020). These supportive approaches have been shown to be beneficial as adjuvant therapies in COVID-19 patients.

Repurposed drugs with proofs of antiviral effects for other viral infections have been tried. However, the efficacy and safety of these drugs in COVID-19 patients are unclear. So far, only remdesivir was approved by FDA for the compassionate use in severe infected COVID-19 patients (Beigel et al., 2020). Other anti-viral drugs, such as arbidol, chloroquine phosphate and ritonavir, did not exhibit efficacy in randomized, placebo-controlled trials in COVID-19 patients. Regardless, it seems reasonable that antiviral therapy might be adopted to patients with high risk factors as early as possible rather than wait for severe manifestation of the disease.

Other therapeutic options including organ support, glucocorticoid therapy, nutritional support have been applied to COVID-19 clinical treatment without much knowledge of their efficacy. However, among all current treatments mentioned above, little attention has been paid to the abnormal humoral metabolism and pulmonary edema, which is a key factor threatening patients’ lives.

## Putative Drug Targets for Pulmonary Edema in COVID-19 Patients

Ion channels, AQPs, RAS, bradykinin and hyaluronic acid are factors influencing the pulmonary edema. The abnormal humoral metabolism in lungs and the resulting pulmonary edema have become the main life-threatening factors in COVID-19 patients. Thus, the relieve of pulmonary edema should be one of the critical concerns in terms of the treatment of COVID-19 patients. Consequently, drugs that can normalize humoral metabolism should be clinically evaluated for their use in the treatment of COVID-19 patients (Table 1).

**TABLE 1:** Potential drugs for normalizing humoral metabolism.

Drugs	Targets	Functions	References
Terbutaline	ENaC	β_2_-adrenergic agonist	Minakata et al. (1998)
Salbutamol			Sartori et al. (2002)
Amiloride		Prototypic inhibitor of ENaC	Bull and Laragh (1968), Maronde et al. (1983), Adil et al. (2020), Cure and Cumhur Cure (2020)
Furosemide	NKCC	NKCC inhibitor	Pickkers et al, (1997), Solymosi et al. (2013)
Glibenclamide	CFTR	CFTR inhibitor	Solymosi et al. (2013)
CFTR_inh-172_ inhibitor			
MCTR1	Na^+^ channel and Na-K-ATPase	Activate the sodium channel and Na-K-ATPase	Han et al, (2020)
PCTR1			Zhang et al. (2020c)
protectin DX			Zhuo et al. (2018)
Resolving D1		Stimulate AFC through alveolar epithelial sodium channel, Na-K-ATPase via ALX/cAMP/PI3K pathway	Wang et al. 2014
K_Ca3.1_ (1-EBIO)	K^+^ channel	K^+^ channel openers	Fleckenstein (1977); Nayler and Dillon (1986)
K_ATP_ (minoxidil)			
Dexmedetomidine	AQP	Regulate AQP expression	Jiang et al. (2015)
Lipoxin A4 (LXA4)			Shi et al. (2018)
Losartan	AT1	AT1 receptor blockers	Yang et al. (2020a); Liu et al. (2020); Richardson et al. (2020)
Valsartan			
Icatibant	Bradykinin	Bradykinin antagonist	Rasaeifar et al. (2020)
Raloxifene			
Sildenafil			
Cefepime			
Cefpirome			
Imatinib			
Ponatinib			
Abemaciclib			
Entrectinib			
Glucocorticoids	ENaC, cytokines	Regulate ENaC expression and impact cytokines	Chen et al. (1999); Chow et al. (1999); de la Rosa et al. (1999), Otulakowski et al. (1999); Sayegh et al. (1999); Itani et al. (2001); Zhang et al. (2007); Ahmed and Hassan (2020)

### Targeting Sodium Channels and Pumps

Since the inhibition of ENaC induces pulmonary edema formation, targeting ENaC is rational in order to enhance fluid clearance from the alveoli. Studies showed that ENaC activators or stimulators can regulate ENaC-dependent fluid absorption in alveolar and pulmonary edema (Fronius, 2013). The activation of β-adrenergic receptor, especially β_2_ (Mutlu et al., 2004), was found to stimulate Na+ and fluid reabsorption. It was observed that the expression of ENaC and Na+/K+-ATPase in primary alveolar type II cells from rat lungs increased responding to terbutaline (Minakata et al., 1998). Inhalation or infusion of salbutamol, a β_2_-adrenergic agonist, reduced the incidences of pulmonary edema (Sartori et al., 2002) and was found to be beneficial in ARDS patients (Perkins et al., 2006). Glucocorticoids were shown to have the ability of inducing *de novo* synthesis of ENaC (Chow et al., 1999; Otulakowski et al., 1999; Sayegh et al., 1999) and affecting ENaC regulatory pathway via serum and glucocorticoids-inducible kinase-1 (SGK-1) (Chen et al., 1999; de la Rosa et al., 1999; Itani et al., 2001; Zhang et al., 2007). However, clinical evidence showing that glucocorticoids can reduce pulmonary edema by regulating fluid absorption in alveolar and glucocorticoids is missing. Potentially, glucocorticoids can be used as anti-inflammatory drugs in ARDS and pulmonary edema as well as in COVID-19 patients. Amiloride, a prototypic inhibitor of ENaC, might also have a potential in treating COVID-19 patients. Amiloride was shown to induce the reduction of ACE2 expression in bronchial and alveolar epithelial cells (Adil et al., 2020) and can counteract the low cytosolic pH which has been observed in COVID-19 patients by acting on Na+/H+ exchanger (Cure and Cumhur Cure, 2020). Since hypokalemia is a major issue in severe COVID-19 patients (Lippi et al., 2020), amiloride with its potassium-sparing diuretic activity (Bull and Laragh, 1968) can potentially be used to restore normal serum potassium concentrations (Maronde et al., 1983). However, as we discussed above, the inhibition of ENaC can also induce pulmonary edema and further studies are required to determine the potential use of ENaC inhibitors for COVID-19 treatment.

CFTR and NKCC inhibitors show promise in treating pulmonary edema. Furosemide, a NKCC inhibitor, has been acknowledged as first-line therapeutic drug for pulmonary edema all the time (Pickkers et al., 1997; Solymosi et al., 2013). CFTR inhibitors, like glibenclamide and CFTR_inh-172_ inhibitor, distinctly reduced absorptive alveolar fluid transport (Solymosi et al., 2013).

Since pulmonary edema can be promoted by decreased expression of alveolar Na-K-ATPase (Woods et al., 2015), drugs or compounds which activate the sodium channel and Na-K-ATPase may be putative therapeutics. Studies have shown that MCTR1 (Han et al., 2020), PCTR1 (Zhang et al., 2020) and protectin DX (Zhuo et al., 2018), endogenously produced lipid mediators, can effectively improve PFC, ameliorate morphological damage, reduce lung inflammation, and increase sodium channel and Na-K-ATPase expression and activity *in vivo* and *in vitro* in lipopolysaccharide (LPS)-induced ARDS rats model. Resolving D1 (Wang et al., 2014), generated from ω-3 fatty docosahexaenoic acids, and ursodeoxycholic acid (Niu et al., 2019) can stimulate AFC and Na-K-ATPase in LPS-induced pulmonary edema via alveolar epithelial sodium channel and ALX/cAMP/PI3K pathway, respectively.

### Targeting Potassium Channels

Potassium channels modulate the expression of ENaC. It has been reported that transepithelial ion transport in alveolar monolayers can be activated by K^+^ channel openers *in vitro* under physiological conditions (Leroy et al., 2006). K_Ca3.1_ (1-EBIO) and K_ATP_ (minoxidil) channel openers can greatly recover AFC in mice intratracheally administrated verapamil, which is the first generation of the phenylalkylamine class of calcium channel antagonists (Fleckenstein, 1977; Nayler and Dillon, 1986), suggesting that K^+^ channel openers might be potential drugs for treating pulmonary edema (Han et al., 2010).

### Targeting Aquaporins and Transient Receptor Potential Ion Channels

AQP-5 plays a significant role in pulmonary edema and decreased expression of AQP-5 has been observed during the disease process. Dexmedetomidine can upregulate AQP-1 and AQP-5 expression in rats with acute lung injury induced by LPS and thus induce pulmonary edema (Jiang et al., 2015). Lipoxin A4 (LXA4) can stabilize the permeability of pulmonary microvascular endothelial cell by regulating the expression of AQP-5 and MMP-9, and reduce alveolar fluid exudation (Shi et al., 2018).

TRPs are essential for the respiratory system and pulmonary edema, in which TRPA1, TRPV1 and TRPV4 are the most important. Inhibiting these TRPs may benefit the treatment of pulmonary edema. A recent review has summarized the effects of TRPs in pulmonary chemical injuries, which includes the representative TRPA1, TRPV1 and TRPV4 antagonists which have participated in preclinical and clinical studies (Achanta and Jordt, 2020) (Supplementary Tables S1–S3).

### Targeting Renin Angiotensin System and Bradykinin

RAS plays a significant role in ARDS as well as pulmonary edema processes. The activities of the molecules in RAS are ruled by dynamic changes responding to an injury. As the activation of AT1 receptor promotes the pulmonary edema, AT1 receptor blockers (ARBs) like losartan, valsartan may be effective in decreasing pulmonary edema. However, preliminary reports showed that some ACE inhibitors and ARBs have no significant clinical benefits in treating COVID-19 (Richardson et al., 2020), while others showed protective effects among patients with pre-existing hypertension (Yang et al., 2020; Liu et al., 2020). The likelihood of hypertensive patients developing COVID-19, who were treated by ARBs, was reported to decrease by 76% (Yan et al., 2020). Moreover, exogenous delivery of Ang (1–7) was shown to play a part in reducing inflammation and improving pulmonary function in ARDS models (Wosten-van Asperen et al., 2011). Recombinant ACE2 was also reported to be a potential therapy in the clinical study of ARDS, which can lead to rapid decrease of plasma Ang II level and IL-6 expression. (Imai et al., 2007; Zhang and Baker, 2017).

Raising evidences suggest that the effect of kinins on bradykinin receptor triggers the inflammatory responses, which have been observed in patients with COVID-19. Consequently, the use of bradykinin antagonists is supposed to be regarded as a strategy for COVID-19 treatment interventions. Currently, only icatibant has been approved as bradykinin antagonist for clinical treatment, and relevant studies have revealed that cefepime, cefpirome, imatinib, raloxifene, sildenafil, ponatinib, abemaciclib and entrectinib may also act as prospective non-selective bradykinin antagonists and have potential for treatment of COVID-19 (Rasaeifar et al., 2020). However, further researches into the mode of action, efficacy and safety of these drugs are required.

### Targeting Hyaluronic Acid and Proteoglycans

Accumulation of HA can directly induce ARDS and pulmonary edema. Thus, promoting the degradation of HA may be significant in the recovery process. HA is synthesized by HAS2. However, so far, no effective inhibitors have been developed against HAS2. HA is degraded by hyaluronidases encoded by HYAL1 and HYAL2, whose activity depends on CD44, an HA receptor (Harada and Takahashi, 2007). CD44 inhibition reduces the IL-2 induced vascular leakage syndrome, revealing that CD44 may act as a potential target in COVID-19 treatment. Nonetheless, little attention has been paid to CD44 in COVID-19 treatment. Further studies may determine if CD44 inhibitors can be of use to in COVID-19.

It has already been shown that the dysregulated release of cytokines is one of the key factors behind poor outcomes in COVID-19 patients. These cytokine storms can be treated with steroids, IL-1 antagonists, TNF inhibitors, and Janus kinase inhibitor (JAK) inhibitors (McCreary and Pogue, 2020). Fedratinib, an FDA approved JAK2 inhibitor, may be used to reduce the mortality associated with hyperinflammation by suppressing the production of several Th17 cytokines (i.e., IL1b and TNF-alpha, IL21, IL22, IL17) and the formation of pulmonary edema in combination with anti-viral drugs (Yang et al., 2020e). Glucocorticoids, especially dexamethasone, have already been applied in the clinical treatment of COVID-19. It not only can induce *de novo* synthesis of ENaC and affect ENaC regulatory pathway, but also has an impact on cytokines. A recent review suggested that low-to-moderate doses of dexamethasone may lower the mortality rate in patients with severe infections (Ahmed and Hassan, 2020) and the latest finding indicated that the neutrophil-to-lymphocyte ratio determines the clinical efficacy of corticosteroid therapy in COVID-19 patients, as a neutrophil-to-lymphocyte ratio >6.11 associating with lower mortality in patients on corticosteroids (Cai et al., 2021). Moreover, other drugs which can regulate immune system like hydroxychloroquine and azithromycin may also show effects in treating SARS-COV-2-induced pulmonary edema and their effectiveness in treating SARS-COV-2 infections should be further investigated.

## Natural Compounds and Traditional Chinese Medicines for the Treatment of Pulmonary Edema in COVID-19

Besides chemical drugs and compounds we discussed above, natural compounds and TCMs also possess promising antiviral effects against SARS-CoV-2 and had notably contribution in curing COVID-19, especially in alleviating pulmonary edema and preventing the disease development from mild to severe. In TCM theory, evils of COVID-19 are derived from cold-dampness, whose core pathogenesis are “toxin” and “dampness”. “Toxin” means various pathogenic microorganism and inflammatory storm presenting in infected patients, and “dampness” means the abnormal humoral metabolism like inflammatory exudation. Here, we summarized several natural compounds and TCM formulas which are very likely to be potential drugs or have already shown predominant efficacy of COVID-19 patients.

### Natural Compounds and Their Effects on Syndrome Coronavirus 2 Infections

Extensive studies have been conducted to identify the antiviral and pulmonary edema reducing efficacy of natural compounds, some of which have already been tested specifically against SARS-CoV and SARS-COV-2. Some natural compounds along with their antiviral and reducing pulmonary edema mechanisms are shown in Table 2.

**TABLE 2:** Summary of potential natural compounds against COVID-19.

Plant	Compound	Structure	Antiviral and reducing pulmonary edema mechanisms	References
*Stephania Japonica*	Cepharanthine	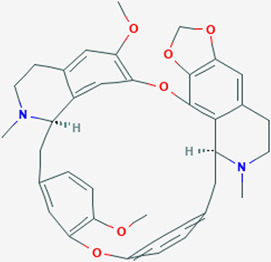	ACE inhibitor	Fan et al. (2020), Rogosnitzky et al. (2020)
*Rheum palmatum*	Emodin	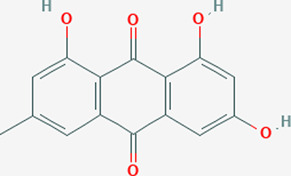	Blocks the binding of S protein to ACE2	Ho et al. (2007)
Black tea	Theaflavin	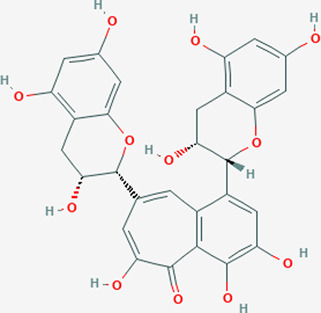	Inhibits RdRp activity	Lung et al. (2020)
*Atractylodes macrocephala*	Atractylonolide-I	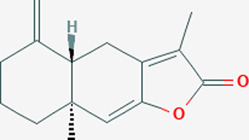	Inhibits the formation of IL-6 and TNF-α	Ji et al. (2014)
*Astragalus propinquus*	Astragaloside-IV	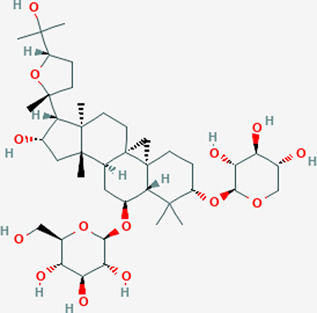	Activates ACE2-Ang-(l–7)-Mas pathway	Wang (2015), Qu (2019)
*Salvia miltiorrhiz*	Cryptotanshinone	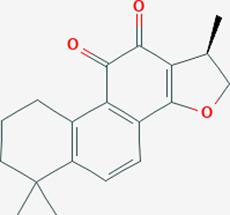	Induces the synthesis of cGMP and NO in cells and activating NO/cGMP pathway	Zhou et al. (2006)

Natural compounds can inhibit the binding between the virus and the ACE2 receptor of host cells. Cepharanthine, a bisbenzylisoquinoline alkaloid derived from tubers of *Stephania Japonica,* was shown to have a wide-spectrum inhibitor of pan-β-coronavirus (Fan et al., 2020; Rogosnitzky et al., 2020). Emodin, an anthraquinone compound from genus Rheum and Polygonum, can markedly prevent the binding of S protein and ACE2 in the study of SARS-CoV (Ho et al., 2007).

RNA-dependent RNA polymerase (RdRp) is a crucial protease that catalyzes RNA replication from RNA templates and is an appealing therapeutic target. Theaflavin from black tea was found to present a lower binding energy when it docks in the catalytic pocket of SARS-CoV-2 RdRp. Thus, it could be a potential RdRp inhibitor for SARS-CoV-2 (Lung et al., 2020).

Inhibiting the inflammatory storm can effectively alleviate pulmonary edema. Atractylenolide-I, the active component of atractylodes, could minimize the formation of IL-6 and TNF-α (Ji et al., 2014), inhibiting the generation of inflammatory cytokines causing inflammatory response, thus lowering the possibility of developing pulmonary edema.

As RAS plays an important role in PFC and AFC, astragaloside IV from *Astragalus propinquus* is able to protect kidney and respiratory by activating the ACE2-Ang-(1–7)-Mas pathway in RAS and improving ACE2, Ang-(1–7), Mas level (Wang et l., 2015; Qu, 2019).

When SARS-COV-2 invades, vascular endothelial cells are damaged, causing insufficient arterial flow and minimal thrombus. Elevated pulmonary venous pressure leads to faster fluid infiltration into the interstitial lung than the ability of the pulmonary lymphatic vessel to drain away fluid, resulting in pulmonary edema. Improving the blood circulation can advance the oxygen supply for organs, accelerate the absorption of fluid and eventually improve pulmonary edema. Zhou et al. (2006) reported that the cryptotanshinone in *Salvia miltiorrhiz* can inhibit the synthesis of cGMP and NO in vein endothelial cells, activate NO/cGMP pathway and improve the blood circulation.

### Traditional Chinese Medicines and COVID-19 Infection

Multiple TCMs have been already clinical applied for COVID-19 in China and achieved high recovery rate. Some of these TCMs along with their constituent and antiviral and reducing pulmonary edema mechanisms are shown in Table 3. *Lian-Hua-Qing-Wen capsules* significantly affect virus morphology, inhibit the SARS-CoV-2 replication with the IC_50_ value of 411.2 μg/ml, reduce pro-inflammatory cytokines production at the mRNA level, and show anti-inflammatory effect *in vitro* (Runfeng et al., 2020). It has been revealed that COVID-19 patients treated with *Lian-Hua-Qing-Wen* capsules for 14 days resulted in a considerably higher recovery rate of 91.5%, a dramatically shorter median time to symptom recovery of 7 days than the control group, which applied conventional treatment (Hu et al., 2021). Moreover, the constituent Ephedra can trigger bronchodilation, relieve breathing disorders and alleviate pulmonary edema (Zhang et al., 2018). The toad venom injection can significantly improve the pulmonary function of COVID-19 patients by regulating PaO2/FiO2 and ROX index and thus alleviate pulmonary edema. As reported, PaO2/FiO2 and ROX index of patients receiving conventional treatment combined with toad venom injection (20 ml/day) improved significantly (−111.30 to −35.90 for PaO2/FiO2 and −7.56 to −2.94 for ROX). Meanwhile, the number of patients in the treatment group presenting improved PaO2/FiO2 and ROX index was higher than that of the control group (95.2% vs. 68.4% and 73.7%). Moreover, the peripheral blood mononuclear lymphocyte of COVID-19 patients was also greatly improved, from 0.91 ± 0.54 to 1.24 ± 0.67 after being treated for a week, while there was no obvious change in control group (Hu et al., 2020). The Liu Shen capsule, of which pharmacodynamic component is also toad venom, was shown to have antiviral and anti-inflammatory activity against SARS-CoV-2 in vitro, as it can inhibit the replication of SARS-CoV-2 in Vero E6 cells, reduce inflammatory cytokines production at the mRNA levels and suppress the NF-κB signaling pathway to downregulate the expression of cytokines (Ma et al., 2020a). *Qing-Fei-Pai-Du* Decoction, which is officially recommended for the treatment of COVID-19 patients as mentioned in the guideline issued by NHC (Trial 7th edition) (PRC, 2020), has an effective rate higher than 90% (2020) and can mediate the inflammatory storm induced by COVID-19 (Yang et al., 2020b), regulate the innate immune, cytokine activities (IL-17, NF-κB, TNF etc.), cell growth and death, as well as the degradation of damaged cells (Zhao et al., 2020). Moreover, as a retrospective multicenter cohort study reported, early treatment with *Qing-Fei-Pai-Du* Decoction associated with better outcomes, faster recovery, and a shorter duration of hospital stay (Shi et al., 2020a). *Xue-Bi-Jing* injection is also widely applicated in treating COVID-19 patients and by adding it based on the routine anti-infective therapy, the 28-day mortality of patients with severe pneumonia could be reduced by 8.8%, greatly improving pneumonia severity index (from 93.18 ± 23.17 to 52.18 ± 30.53) (Ma et al., 2020b). *Xue-Bi-Jing* injection may act in COVID-19 by anti-inflammatory, anticoagulation, immune regulation, vascular endothelial protection, anti-oxidative stress and other mechanisms (Li et al., 2021).

**TABLE 3:** Summary of potential TCM formulae against COVID-19.

TCM formulae	Constituent	Antiviral and reducing pulmonary edema mechanisms	Clinical efficacy	References
*Lian-Hua-Qing-Wen* Capsule	Forsythiae Fructus, Lonicerae Japonicae Flos, Ephedrae Herba, Armeniacae Semen, Amarum, Isatidis Radix, Dryopteridis Crassirhizomatis Rhizoma, Houttuyniae	Inhibits the replication of SARS-CoV-2, affects virus morphology, exert anti-inflammatory activity and triggers bronchodilation	Combined treatment had higher recovery rate (91.5% vs. 82.4%, *p* = 0.022), a dramatically shorter median time to symptom recovery (7 vs. 10 days, *p* < 0.001), as well as a remarkably shorter time to recovery of fever (2 vs. 3 days), fatigue (3 vs. 6 days) and coughing (7 vs. 10 days) (*p* < 0.001 for all)	Zhang et al. (2018), Luo et al. (2020), Runfeng et al. (2020)
	Herba, Pogostemonis Herba, Rhei Radix et Rhizoma, Rhodiolae Crenulatae, Radix et Rhizoma, Glycyrrhizae Radix et Rhizoma and Gypsum Fibrosum			
Qing-Fei-Pai-Du *Decoction*	Astragali Radix, Bupleuri Radix, Ephedrae Herba, Armeniacae Semen Amarum, Gypsum Fibrosum, Coicis Semen, Trichosanthis Pericarpium, Platycodonis Radix, Menthae Haplocalycis Herba, Scutellariae Radix, Glycyrrhizae Radix et Rhizoma, Lonicerae Japonicae Flos, and Artemisiae Annuae Herba	Intervenes the inflammatory storm and triggers bronchodilation	Has an effective rate higher than 90% and early treatment with Qing-Fei-Pai-Du Decoction can result better outcomes, faster recovery, and a shorter duration of hospital stay	Shi et al. (2020a), Yang et al. (2020b)
*Toad venom* Injection	Toad venom	Improving PaO2/FiO2 and ROX index	Improves the PaO2/FiO2 and ROX index (*p* < 0.001, 95% CI, −111.30 to −35.90 for PaO2/FiO2; *p* < 0.001, 95% CI, −7.56 to −2.94 for ROX) by 95.2%	Hu et al. (2020)
*Liu Shen* Capsule	Bezoar, Musk, venom toad, pearl, realgar, and borneol	Inhibiting the replication of SARS-CoV-2, reducing inflammatory cytokines production at the mRNA levels and suppressing the NF-κB signaling pathway to downregulate the expression of cytokines *in vitro*	Improves respiratory function and lymphocyte count (similar to the *Toad venom* Injection)	Ma et al. (2020a)
*Xue-Bi-Jing* Injection	Carthami Flos, Paeoniae Radix Rubra, Chuanxiong Rhizoma, Salviae Miltiorrhizae, Radix et Rhizoma, Angelicae Sinensis Radix	Anti-inflammatory, anti-coagulation, immune regulation, vascular endothelial protection, anti-oxidative stress and other mechanisms	The 28-day mortality of patients with severe pneumonia could be reduced by 8.8%, significantly improving pneumonia severity index (from 93.18 ± 23.17 to 52.18 ± 30.53)	Ma et al. (2020b), Li et al. (2021)

## Conclusion

The abnormal humoral metabolism and pulmonary edema contribute to the severity of symptoms and fatality of COVID-19 patients. Decreased expression of alveolar Na-K-ATPase, dysregulation of sodium and potassium channels, aquaporins, and renin angiotensin system, and abnormal metabolism of bradykinin and hyaluronic acid as well as cytokine inflammatory storm all lead to ARDS and pulmonary edema. These in turn, lead to severe lung damage in COVID-19 patients. Existing drugs and inhibitors targeting the components of humoral metabolism may serve as potential treatments for COVID-19 and should be further investigated. In addition, natural compounds and TCMs which generally have multiple targets should also be investigated, both in terms of their efficacy and safety. Focusing on decreasing the formation of body fluid in lung or promoting the absorption of body fluid can contribute to a decrease in lung damage and decreased mortality in COVID-19 patients. Therefore, drugs targeted at the humoral mechanisms might turn out to be highly effective against SARS-COV-2 infections.
