# Supplementary material for: Pulmonary Edema in COVID-19 Patients: Mechanisms and Treatment Potential
Source: Front Pharmacol. 2021 Jun 7;12:664349. doi: 10.3389/fphar.2021.664349 (PMC8215379; doi:10.3389/fphar.2021.664349)
Supplement: Supplementary file 1 [file DataSheet1.docx]

## **Supplements**

The three supplementary tables are antagonists of three transient receptor potentials (TRPs), including TRPA1, TRPV1, TRPV4, that we have discussed in the review and we consider these antagonists as potential drugs for COVID-19 treatment.

Supplement Table 1. TRPA1 antagonists

| Name | Structure | Activity species | Selectivity | IC50 value | Refs. |
| --- | --- | --- | --- | --- | --- |
| HC‐030031  2‐(1,3‐dimethyl‐2,6‐dioxo‐1,2,3,6‐tetrahydro‐7H‐purin‐7‐yl)‐N‐(4‐isopropylphenyl)acetamide | 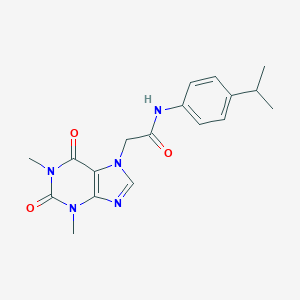 | Mouse, rat, and human | Selective | 6.2 μM (human);  7.6 μM (rat) | (McNamara et al., 2007) |
| AP18  4‐(4‐chlorophenyl)‐3‐methyl‐3‐buten‐2‐one oxime | 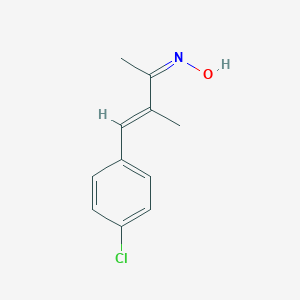 | Mouse, rat, and human | Selective | 3.1 μM (human);  8.8 μM (rat);  4.5 μM (mouse) | (Petrus et al., 2007) |
| ChemBridge‐5861528  N‐(4‐butan‐2‐ylphenyl)‐2‐(1,3‐dimethyl‐2,6‐dioxopurin‐7‐yl)acetamide | 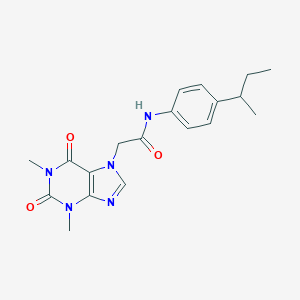 | Rat and human | Selective | 4.9 μM (human);  0.3 μM (rat) | (Wei et al., 2009) |
| A‐967079  (1E,3E)‐1‐(4‐fluorophenyl)‐2‐methyl‐1‐pentene‐3‐one oxime | 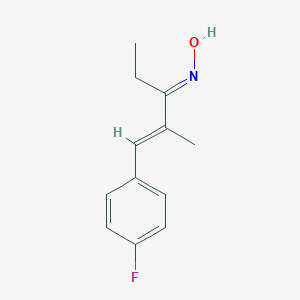 | Mouse, rat, and human | Selective | 0.067 μM (human);  0.289 μM (rat) | (McGaraughty et al., 2010) |
| AMG0902 | 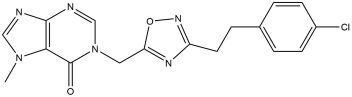 | Rat and human | Selective | 0.071 μM (rat);  0.131 μM | (Lehto et al., 2016; Schenkel et al., 2016) |

Supplement Table 2. TRPV1 antagonists

| Name | Structure | Activity species | Selectivity | IC50 value | Refs. |
| --- | --- | --- | --- | --- | --- |
| Capsazepine  N‐[2‐(4‐chlorophenyl)ethyl]  ‐1,3,4,5‐tetrahydro‐7,8‐dihydroxy‐2H‐2‐benzazepine‐2‐carbothioamide | 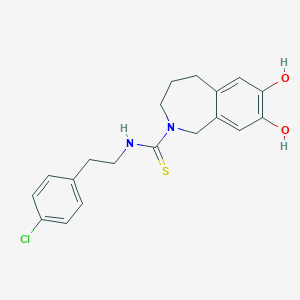 | Rat, and human | Selective | 420–562 nM (rat)  58 nM (human) | (Bevan et al., 1992) |
| SB‐705498  N‐(2‐bromophenyl)‐N′‐[(3R)‐1‐[5‐(trifluoromethyl)‐2‐pyridinyl]‐3‐pyrrolidinyl]‐urea | 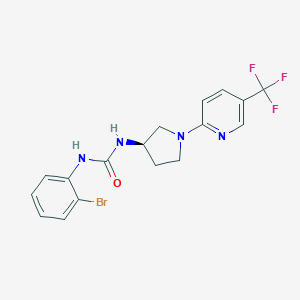 | Mouse, rat, and human | Selective | 0.9 nM (rat);  3–6 nM (human) | (Gunthorpe et al., 2007) |
| AMG‐517  N‐[4‐[[6‐[4‐(trifluoromethyl)phenyl]‐4‐pyrimidinyl]oxy]‐2‐benzothiazolyl]‐acetamide | 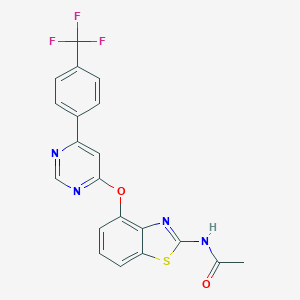 | Mouse, rat, dog, monkey and human | Selective | 0.5 nM (rat);  0.9 nM (human) | (Blum et al., 2010) |
| ABT‐102  (R)‐1‐(5‐tert‐butyl‐2,3‐dihydro‐1H‐inden‐1‐yl)‐3‐(1H‐indazol‐4‐yl)urea | 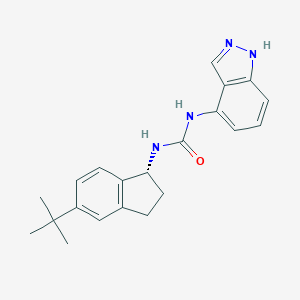 | Rat, and human | Selective | 1–16 nM (rat);  5–7 nM (human) | (Surowy et al., 2008) |
| MK‐2295/NGD8243  N‐[4‐(trifluoromethyl)phenyl]‐7‐[4‐(trifluoromethyl)pyridin‐3‐yl]quinazolin‐4‐amine | 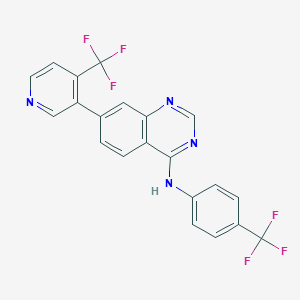 | Mouse, rat and human | Selective | 0.3–6 nM (human) | (Information, 2020) |
| AZD‐1386  (S)‐N‐(1‐(4‐(tert‐butyl)phenyl)ethyl)‐2‐(6,7‐difluoro‐1H‐benzo[d]imidazol‐1‐yl)acetamide | 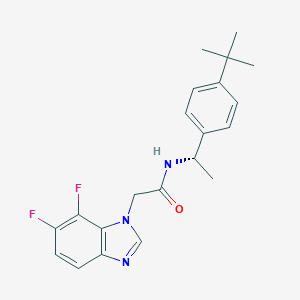 | Human | Selective | 25 nM | (Krarup et al., 2011; Quiding et al., 2013) |
| JTS‐653  (3S)‐3‐(hydroxymethyl)‐4‐(5‐methylpyridin‐2‐yl)‐N‐[6‐(2,2,2‐trifluoroethoxy)pyridin‐3‐yl]‐3,4‐dihydro‐2H‐benzo[b][1,4]oxazine‐8‐carboxamide | 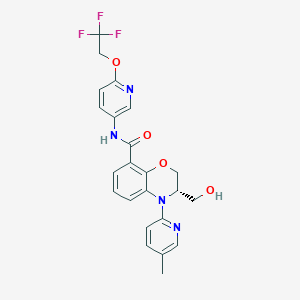 | Mouse, rat and human | Selective | 0.347 nM (rat);  0.32 nM (human) | (Kitagawa et al., 2012) |
| Mavatrep (JNJ‐39439335)  trans‐2‐(2‐{2‐[2‐(4‐trifluoromethyl‐phenyl)‐vinyl]‐1H‐benzimidazol‐5‐yl}xx‐phenyl)‐propan‐2‐ol | 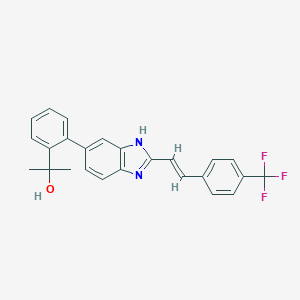 | Human | Selective | 4.6 nM | (Parsons et al., 2015; Manitpisitkul et al., 2018) |

Supplement Table 3. TRPV4 antagonists

| Name | Structure | Activity species | Selectivity | IC50 value (human) | Refs. |
| --- | --- | --- | --- | --- | --- |
| Ruthenium red |  | Rat human and porcine | Nonselective | 14 nM | (Nilius et al., 2004; Vincent et al., 2009) |
| RN‐1734  (2,4‐dichloro‐N‐isopropyl‐N‐(2‐isopropylaminoethyl)benzene sulfonamide) | 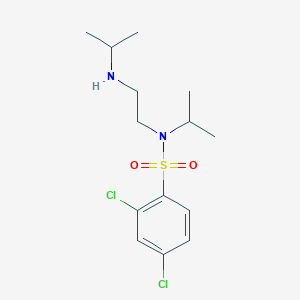 | Mouse, rat and human | Selective | 2.3 μM | (Vincent et al., 2009) |
| GSK2193874  (3‐(1,4′‐bipiperidin‐1′‐ylmethyl)‐7‐bromo‐N‐(1‐phenylcyclopropyl)‐2‐[3‐(trifluoromethyl)phenyl]‐4‐quinolinecarboxamide) | 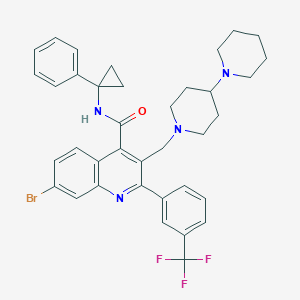 | Rat, mouse, canine and human | Selective | 40 nM | (Thorneloe et al., 2012; Yin et al., 2016; Cheung et al., 2017) |
| GSK2798745  (1‐(((5S,7S)‐3‐(5‐(2‐hydroxypropan‐2‐  yl)pyrazin‐2‐yl)‐7‐methyl‐2‐oxo‐1‐oxa‐3‐  azaspiro[4.5]decan‐7‐yl)methyl)‐1H‐benzo[d]  imidazole‐6‐carbonitrile) | 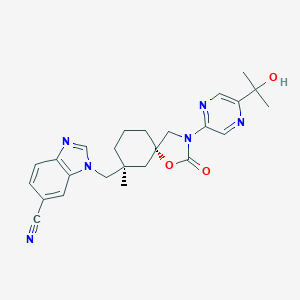 | Mouse, rat, human, dog and monkey | Selective | 1.8 nM | (Brooks et al., 2019b; Goyal et al., 2019) |
| GSK3395879  (4‐(((3S,4R)‐1‐((2‐cyano‐4‐(trifluoromethyl)phenyl)sulfonyl)‐4‐hydroxy‐4‐(hydroxymethyl)pyrrolidin‐3‐yl)oxy)‐2‐fluorobenzonitrile) | 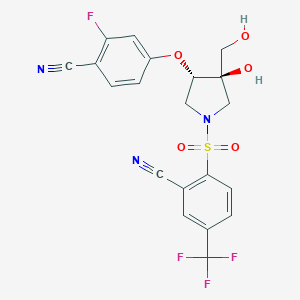 | Human and rat | Selective | 1 nM | (Brnardic et al., 2018) |
| GSK3491943 | 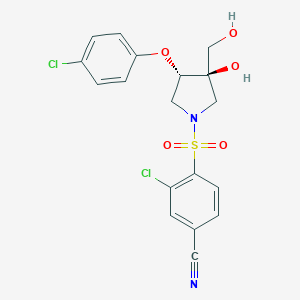 | Human and rat | Selective | 3.2 nM | (Pero et al., 2018) |
| GSK3527497 | 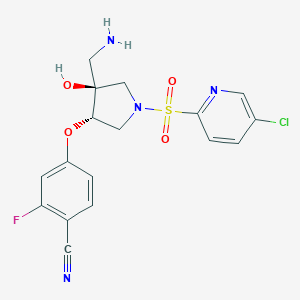 | Human and rat | Selective | 12 nM | (Brooks et al., 2019a) |

## **reference**

Bevan, S., Hothi, S., Hughes, G., James, I.F., Rang, H.P., Shah, K., et al. (1992). Capsazepine a competitive antagonist of the sensory neurone excitant capsaicin. *Br. J. Pharmacol.* 107(2)**,** 544-552.

Blum, C.A., Caldwell, T., Zheng, X., Bakthavatchalam, R., Capitosti, S., Brielmann, H., et al. (2010). Discovery of novel 6,6-heterocycles as transient receptor potential vanilloid (TRPV1) antagonists. *J Med Chem* 53(8)**,** 3330-3348. doi: 10.1021/jm100051g.

Brnardic, E.J., Ye, G., Brooks, C., Donatelli, C., Barton, L., McAtee, J., et al. (2018). Discovery of Pyrrolidine Sulfonamides as Selective and Orally Bioavailable Antagonists of Transient Receptor Potential Vanilloid-4 (TRPV4). *J Med Chem* 61(21)**,** 9738-9755. doi: 10.1021/acs.jmedchem.8b01317.

Brooks, C.A., Barton, L.S., Behm, D.J., Brnardic, E.J., Costell, M.H., Holt, D.A., et al. (2019a). Discovery of GSK3527497: A Candidate for the Inhibition of Transient Receptor Potential Vanilloid-4 (TRPV4). *J Med Chem* 62(20)**,** 9270-9280. doi: 10.1021/acs.jmedchem.9b01247.

Brooks, C.A., Barton, L.S., Behm, D.J., Eidam, H.S., Fox, R.M., Hammond, M., et al. (2019b). Discovery of GSK2798745: A Clinical Candidate for Inhibition of Transient Receptor Potential Vanilloid 4 (TRPV4). *ACS Med Chem Lett* 10(8)**,** 1228-1233. doi: 10.1021/acsmedchemlett.9b00274.

Cheung, M., Bao, W., Behm, D.J., Brooks, C.A., Bury, M.J., Dowdell, S.E., et al. (2017). Discovery of GSK2193874: An Orally Active, Potent, and Selective Blocker of Transient Receptor Potential Vanilloid 4. *ACS Med Chem Lett* 8(5)**,** 549-554. doi: 10.1021/acsmedchemlett.7b00094.

Goyal, N., Skrdla, P., Schroyer, R., Kumar, S., Fernando, D., Oughton, A., et al. (2019). Clinical Pharmacokinetics, Safety, and Tolerability of a Novel, First-in-Class TRPV4 Ion Channel Inhibitor, GSK2798745, in Healthy and Heart Failure Subjects. *Am J Cardiovasc Drugs* 19(3)**,** 335-342. doi: 10.1007/s40256-018-00320-6.

Gunthorpe, M.J., Hannan, S.L., Smart, D., Jerman, J.C., Arpino, S., Smith, G.D., et al. (2007). Characterization of SB-705498, a potent and selective vanilloid receptor-1 (VR1/TRPV1) antagonist that inhibits the capsaicin-, acid-, and heat-mediated activation of the receptor. *J Pharmacol Exp Ther* 321(3)**,** 1183-1192. doi: 10.1124/jpet.106.116657.

Information, N.C.f.B. (2020). PubChem Compound Summary for CID 56603682. *National Center for Biotechnology Information*.

Kitagawa, Y., Miyai, A., Usui, K., Hamada, Y., Deai, K., Wada, M., et al. (2012). Pharmacological characterization of (3S)-3-(hydroxymethyl)-4-(5-methylpyridin-2-yl)-N-[6-(2,2,2-trifluoroethoxy)pyridin-3-yl]-3,4-dihydro-2H-benzo[b][1,4]oxazine-8-carboxamide (JTS-653), a novel transient receptor potential vanilloid 1 antagonist. *J Pharmacol Exp Ther* 342(2)**,** 520-528. doi: 10.1124/jpet.112.194027.

Krarup, A.L., Ny, L., Astrand, M., Bajor, A., Hvid-Jensen, F., Hansen, M.B., et al. (2011). Randomised clinical trial: the efficacy of a transient receptor potential vanilloid 1 antagonist AZD1386 in human oesophageal pain. *Aliment Pharmacol Ther* 33(10)**,** 1113-1122. doi: 10.1111/j.1365-2036.2011.04629.x.

Lehto, S.G., Weyer, A.D., Youngblood, B.D., Zhang, M., Yin, R., Wang, W., et al. (2016). Selective antagonism of TRPA1 produces limited efficacy in models of inflammatory- and neuropathic-induced mechanical hypersensitivity in rats. *Mol Pain* 12**,** 1744806916677761. doi: 10.1177/1744806916677761.

Manitpisitkul, P., Flores, C.M., Moyer, J.A., Romano, G., Shalayda, K., Tatikola, K., et al. (2018). A multiple-dose double-blind randomized study to evaluate the safety, pharmacokinetics, pharmacodynamics and analgesic efficacy of the TRPV1 antagonist JNJ-39439335 (mavatrep). *Scand J Pain* 18(2)**,** 151-164. doi: 10.1515/sjpain-2017-0184.

McGaraughty, S., Chu, K.L., and Perner, R.J. (2010). TRPA1 modulation of spontaneous and mechanically evoked firing of spinal neurons in uninjured, osteoarthritic, and inflamed rats. *Mol Pain* 6**,** 14. doi: 10.1186/1744-8069-6-14.

McNamara, C.R., Mandel-Brehm, J., and Bautista, D.M. (2007). TRPA1 mediates formalin‐induced pain. *Proc Natl Acad Sci U S A* 104(33)**,** 13525-13530. doi: 10.1073/pnas.0705924104.

Nilius, B., Vriens, J., and Prenen, J. (2004). TRPV4 calcium entry channel a paradigm for gating diversity. *Am J Physiol Cell Physiol* 286(2)**,** C195-205. doi: 10.1152/ajpcell.00365.2003.

Parsons, W.H., Calvo, R.R., Cheung, W., Lee, Y.K., Patel, S., Liu, J., et al. (2015). Benzo[d]imidazole Transient Receptor Potential Vanilloid 1 Antagonists for the Treatment of Pain: Discovery of trans-2-(2-{2-[2-(4-Trifluoromethyl-phenyl)-vinyl]-1H-benzimidazol-5-yl}-phenyl)- propan-2-ol (Mavatrep). *J Med Chem* 58(9)**,** 3859-3874. doi: 10.1021/acs.jmedchem.5b00132.

Pero, J.E., Matthews, J.M., Behm, D.J., Brnardic, E.J., Brooks, C., Budzik, B.W., et al. (2018). Design and Optimization of Sulfone Pyrrolidine Sulfonamide Antagonists of Transient Receptor Potential Vanilloid-4 with in Vivo Activity in a Pulmonary Edema Model. *J Med Chem* 61(24)**,** 11209-11220. doi: 10.1021/acs.jmedchem.8b01344.

Petrus, M., Peier, A.M., Bandell, M., Hwang, S.W., Huynh, T., Olney, N., et al. (2007). A role of TRPA1 in mechanical hyperalgesia is revealed by pharmacological inhibition. *Mol Pain* 3**,** 40. doi: 10.1186/1744-8069-3-40.

Quiding, H., Jonzon, B., Svensson, O., Webster, L., Reimfelt, A., Karin, A., et al. (2013). TRPV1 antagonistic analgesic effect: a randomized study of AZD1386 in pain after third molar extraction. *Pain* 154(6)**,** 808-812. doi: 10.1016/j.pain.2013.02.004.

Schenkel, L.B., Olivieri, P.R., Boezio, A.A., Deak, H.L., Emkey, R., Graceffa, R.F., et al. (2016). Optimization of a Novel Quinazolinone-Based Series of Transient Receptor Potential A1 (TRPA1) Antagonists Demonstrating Potent in Vivo Activity. *J Med Chem* 59(6)**,** 2794-2809. doi: 10.1021/acs.jmedchem.6b00039.

Surowy, C.S., Neelands, T.R., Bianchi, B.R., McGaraughty, S., El Kouhen, R., Han, P., et al. (2008). (R)-(5-tert-butyl-2,3-dihydro-1H-inden-1-yl)-3-(1H-indazol-4-yl)-urea (ABT-102) blocks polymodal activation of transient receptor potential vanilloid 1 receptors in vitro and heat-evoked firing of spinal dorsal horn neurons in vivo. *J Pharmacol Exp Ther* 326(3)**,** 879-888. doi: 10.1124/jpet.108.138511.

Thorneloe, K.S., Cheung, M., Bao, W., Alsaid, H., Lenhard, S., Jian, M.-Y., et al. (2012). An Orally Active TRPV4 Channel Blocker Prevents and Resolves Pulmonary Edema Induced by Heart Failure. *Science Translational Medicine* 4(159)**,** 159ra148.

Vincent, F., Acevedo, A., Nguyen, M.T., Dourado, M., DeFalco, J., Gustafson, A., et al. (2009). Identification and characterization of novel TRPV4 modulators. *Biochem Biophys Res Commun* 389(3)**,** 490-494. doi: 10.1016/j.bbrc.2009.09.007.

Wei, H., Hamalainen, M.M., Saarnilehto, M., Koivisto, A., and Pertovaara, A. (2009). Attenuation of mechanical hypersensitivity by an antagonist of the TRPA1 ion channel in diabetic animals. *Anesthesiology* 111(1)**,** 147-154.

Yin, J., Michalick, L., Tang, C., Tabuchi, A., Goldenberg, N., Dan, Q., et al. (2016). Role of Transient Receptor Potential Vanilloid 4 in Neutrophil Activation and Acute Lung Injury. *Am J Respir Cell Mol Biol* 54(3)**,** 370-383. doi: 10.1165/rcmb.2014-0225OC.
